# Acute Circulatory Collapse and Advanced Therapies in Patients with COVID-19 Infection

**DOI:** 10.14797/mdcvj.1048

**Published:** 2021-12-15

**Authors:** Rishi Thaker, Aayush Shah, Ju Kim, Mahwash Kassi

**Affiliations:** 1 New York Presbyterian-Brooklyn Methodist Hospital, Brooklyn, NY; 2 Houston Methodist DeBakey Cardiology Associates, Houston, TX

**Keywords:** COVID-19, ECMO, LVAD, Impella, mechanical circulatory support, circulatory collapse

## Abstract

In the current era of the COVID-19 pandemic, intensive care patients with COVID-19 often develop respiratory failure and acute respiratory distress syndrome. While less frequent, acute circulatory collapse, with or without respiratory failure, has its own management challenges and nuances. Early identification of acute circulatory collapse requires appropriate imaging, particularly echocardiography, and precise diagnosis of cardiogenic shock using a Swan-Ganz catheter. Escalation to mechanical circulatory support (MCS), such as an intra-aortic balloon pump, Impella, and extracorporeal membrane oxygenation, has been useful in patients with acute circulatory collapse from COVID-19. This condition is associated with high morbidity and mortality, but early recognition of appropriate candidates for specific treatment strategies and escalation to MCS might improve outcomes.

## Introduction

The coronavirus disease 2019 (COVID-19) pandemic has changed the way we practice medicine forever. Since the disease was first described in Wuhan, China, in December 2019, its effects have been catastrophic, claiming millions of human lives.^1^ Although the severe acute respiratory syndrome coronavirus 2 (SARS-CoV-2) virus predominantly affects the respiratory system, it can affect numerous organ systems, and patients with cardiac disease or cardiovascular involvement have the highest risks for long-term hospitalization and mortality.^2^

According to a global literature survey, up to 33% of hospitalized COVID-19 patients develop severe illness in the form of acute respiratory distress syndrome (ARDS).^3^ The case fatality rate among all patients with COVID-19 is higher in patients with pre-existing conditions such as diabetes, hypertension, or obesity but highest in those with known cardiovascular conditions.^1^

## Cardiovascular Manifestations in COVID-19

SARS-CoV-2 is a single-stranded RNA virus that enters the cells by receptor-mediated endocytosis, binding to angiotensin converting enzyme 2 (ACE2) and transmembrane serine proteinase 2 proteins. ACE2 is commonly expressed in lung, heart, and kidney tissue. More than 7.5% of cardiac myocytes express ACE2; acute circulatory collapse is postulated to occur from direct viral involvement of ACE2 receptors or an exaggerated immune response.^4^ In a study of autopsied hearts of COVID patients in Toronto, the SARS-CoV viral RNA was detected in 35% of patients.^5^ Additionally, myocyte injury ensues due to a cytokine storm that causes an imbalance of T-helper cells and hypoxia-induced myocyte injury.^6^ This hyperinflammatory state can cause plaque instability, vascular or myocardial inflammation, coagulopathy, or myocardial suppression.

Cardiovascular manifestations of COVID-19 infection are wide ranging, including myocarditis,^7^ arrhythmias, acute coronary syndromes, stress cardiomyopathy,^8,9^ and heart failure.^10^ Cases of pericardial effusion with cardiac tamponade have been described. In children, a multisystem inflammatory response can occur, leading to severe disease.^11^ Severe complications can result in heart failure and cardiogenic shock, leading to acute circulatory collapse. There is significant morbidity and mortality when this occurs. Early recognition and institution of appropriate medical therapy is key. If medical therapy fails, mechanical circulatory support (MCS) must be considered. In this article, we highlight the nuances of managing acute circulatory collapse in these patients.

Depending on the underlying mechanism of injury, patients may have one of two cardiac presentations: (1) delayed myocardial damage occurring after hyperinflammation, or (2) fulminant myocarditis leading to acute circulatory collapse. In the first scenario, the initial presentation is not cardiac in nature, and myocyte injury is revealed over time by rising troponin and B-type natriuretic peptide and subsequent reduced ejection fraction (EF). The median onset of symptoms is 4 days; in severe cases, death usually occurs within 20 days of onset.^12^ These patients have a hyperinflammatory response, and the rise in troponin correlates with the rise in inflammatory markers, such as interleukin-6 (IL-6), lactate dehydrogenase, ferritin, and D-dimer. Higher troponin levels are correlated with worse survival.^13^ In the second scenario, the initial presentation is usually cardiac in nature, with symptoms such as chest pain or palpitations, and circulatory collapse is rapid.

## Case presentation

### Case 1

A 42-year-old female presented with progressive hypoxia and, on the second day of admission, developed critical COVID-19 bronchopneumonia requiring intubation. She deteriorated into hemodynamic collapse with mild left ventricular (LV) dysfunction and a pericardial effusion with ventricular interdependence and impending cardiac tamponade. A surgical team emergently performed a pericardial window, draining 400 cc of serous fluid. Despite the pericardial window, the patient developed multiorgan failure and progressive hypotension requiring multiple pressors.

Repeat transthoracic echocardiography (TTE) showed progressive biventricular dysfunction (EF ~20%), and hemodynamics by right heart catheterization were consistent with cardiogenic shock. Our team placed an intra-aortic balloon pump (IABP). An Impella device (Abiomed) was considered, but the patient’s anatomy limited its feasibility.

Next, the patient was transferred to our tertiary care center for further management of cardiogenic shock with multiorgan failure. At this stage, venoarterial extracorporeal membrane oxygenation (VA-ECMO) was considered but ultimately rejected because multiorgan failure made the patient extremely high risk and her prognosis was guarded.

Instead, one dose of tocilizumab, an IL-6 blocker, was given for severe LV dysfunction. Despite therapeutic anticoagulation, the patient developed a nonocclusive thrombus in the right leg where the IABP was inserted. Because the patient was anuric, continuous renal replacement therapy (CRRT) was used to reduce fluid overload.

**Figure 1:**
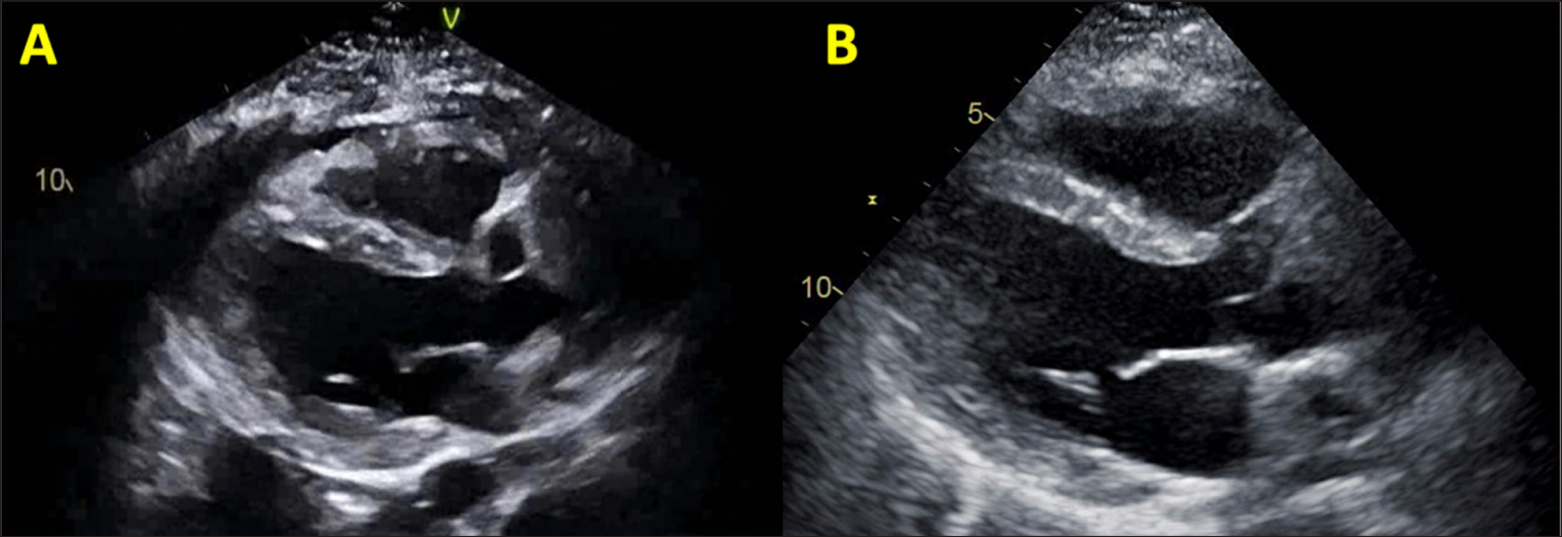
**(A)** Pretreatment echocardiogram (echo) with pericardial effusion, myocardial edema, and depressed left ventricular (LV) and right ventricular (RV) function. **(B)** Post-treatment echo with LV and RV recovery.

On day 6, the patient’s pressor requirements improved, and an echocardiogram showed improvement in her EF. The IABP was removed. Over the course of a few days, she was slowly weaned off pressors and inotropes, and she was extubated on day 9. On day 16, she was discharged on dialysis to a long-term acute care facility and remained stable through a prolonged recovery (***Figure 1***).

### Case 2

**Figure 2:**
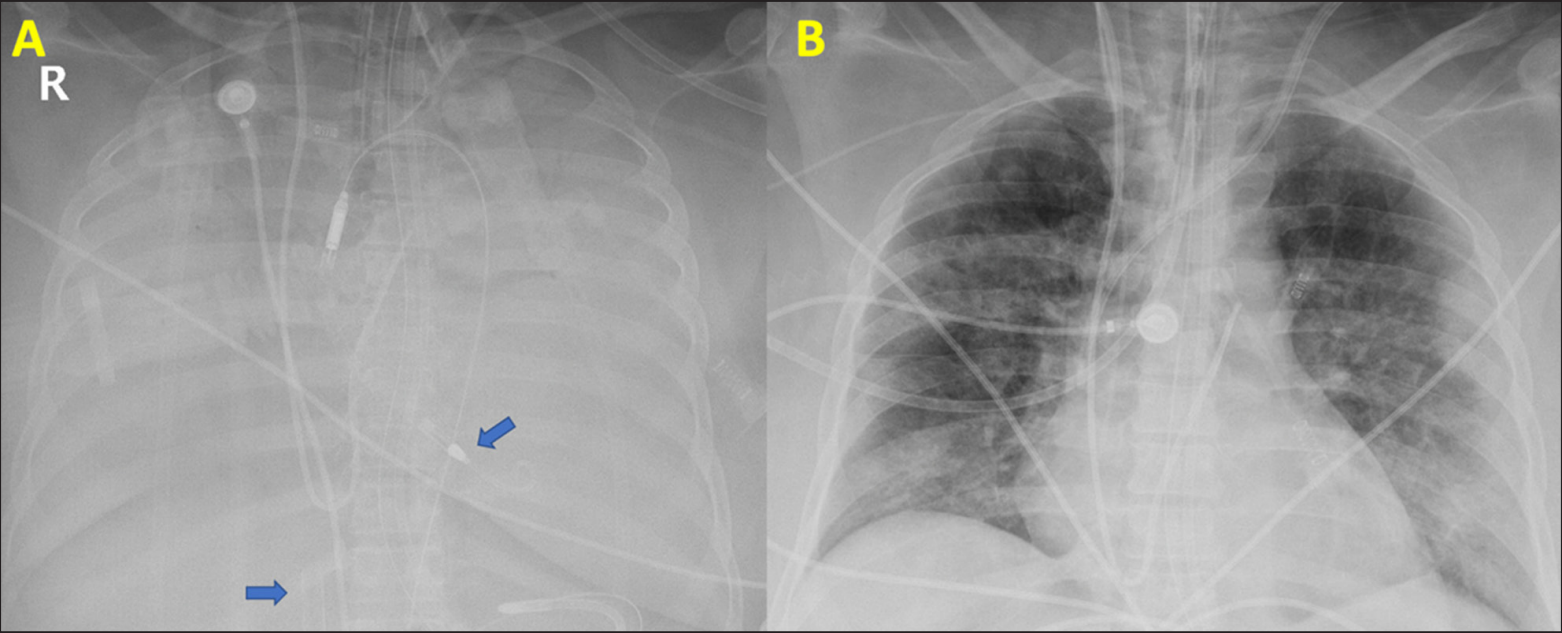
**(A)** Chest x-ray demonstrating severe COVID-19 pneumonia with extracorporeal membrane oxygenation (ECMO) cannula and Impella (arrows). **(B)** Post-decannulation chest x-ray after ECMO and Impella removal.

A 42-year-old female with past medical history significant for obesity presented with COVID-19 bronchopneumonia 6 days after a positive outpatient COVID-19 swab. In the emergency department, she was given 3 L of fluid bolus for severe sepsis and developed flash pulmonary edema requiring emergent intubation. She also developed hemodynamic collapse, requiring inotrope and pressor support, and a TTE demonstrated severely depressed LV ejection fraction (LVEF) of < 10%. Peripheral VA-ECMO was placed, and the patient was transferred to our tertiary care center for further management of fulminant COVID myocarditis with cardiogenic shock. There, the patient was taken to the catheterization lab. There was no significant obstructive coronary artery disease, and an Impella CP (Abiomed) was placed for hemodynamic support. She was started on a high-dose steroid and given one dose of tocilizumab for severe LV dysfunction, two rounds of intravenous immune globulin (2 g/kg divided in 2 days), and continuous renal replacement therapy. She required multiple transfusions due to profuse epistaxis on heparin. On day 11, the patient’s hemodynamics were stable, and there were signs of LV recovery, so she was decannulated. Impella support was continued until complete recovery. At that point, the patient was extubated and continues to recover, as shown in ***Figure 2***.

## Acute Circulatory Collapse

### Risk Factors for Severe Circulatory Collapse

Patients with cardiovascular risk factors are at markedly higher risk of symptomatic and severe COVID-19 disease and account for a large proportion of COVID-19 mortality.^10^ Generally, literature on critical COVID-19 disease revolves around the prevalence of ICU admission, development of shock, and fatality.^10,14,15,16^ These reports typically pool together disparate causes of shock and fail to differentiate septic shock from cytokine storm, obstructive shock from high ventilatory pressures, and cardiogenic shock from fulminant myocarditis.^17^ According to some studies, 25% to 30% of COVID-19 patients admitted to the ICU have cardiogenic shock.^18^ Patients with pre-existing heart failure, coronary artery disease, hypertension, and diabetes are all at higher risk of ICU admission and acute circulatory collapse, but the reason why some patients develop circulatory collapse and others do not is not fully understood.

Acute circulatory collapse can occur in the setting of acute fulminant myocarditis as a result of stress cardiomyopathy from severe cytokine response, or as a sequela of myocardial infarction or worsening heart failure in patients with pre-existing cardiomyopathy. Increased intrathoracic pressure from high ventilation pressures also plays a role.

### Medical Management

The acute management of shock in the ICU remains the same for patients with COVID-19 as for those without. Early recognition of shock and its etiology is the key to success. Patients with incessant arrhythmias, rapidly declining EF, and potential circulatory collapse warrant close monitoring and ICU admission. Right heart catheterization using a Swan-Ganz catheter is instrumental in identifying hemodynamic status and classifying both the extent and etiology of shock.

Although therapy with diuretics and inotropic therapy may be effective, refractory cases of shock warrant consideration for early initiation of MCS. If untreated, cardiogenic shock manifests with systemic hypoperfusion and progressive inflammatory response resulting in multiorgan failure and eventual death. ***Table 1*** outlines important considerations for managing these patients.

**Table 1:** General considerations for intensive care management of patients in acute circulatory collapse. MCS: mechanical circulatory support; RV: right ventricle; LV: left ventricle; CRRT: continuous renal replacement therapy; ARDS: acute respiratory distress syndrome; CO: cardiac output


Hemodynamic assessment	Hemodynamic assessment with Swan-Ganz catheter is at the core of cardiogenic shock management. A Swan-Ganz catheter should be used, particularly if MCS is considered. A central line can assess volume status and mixed venous oxygen saturation. Echocardiography is essential for assessing RV/LV function, CO, and filling pressures.

Fluid management	Avoid hyper- or hypovolemia. In ARDS, hypervolemia may worsen respiratory status. In hyperinflammation with severe cytokine response, functional hypovolemia due to capillary leak and intravascular volume depletion may lead to poor organ perfusion. CRRT may be required to optimize fluid status.

Blood pressure maintenance	Maintain perfusion with vasopressors. Norepinephrine is considered first line. Vasopressin is considered second line.

Adequate cardiac output maintenance	Dobutamine or milrinone may be used to maintain CO. When inotropes fail, early escalation to MCS may be considered as outlined.


### Mechanical Circulatory Support

**Figure 3:**
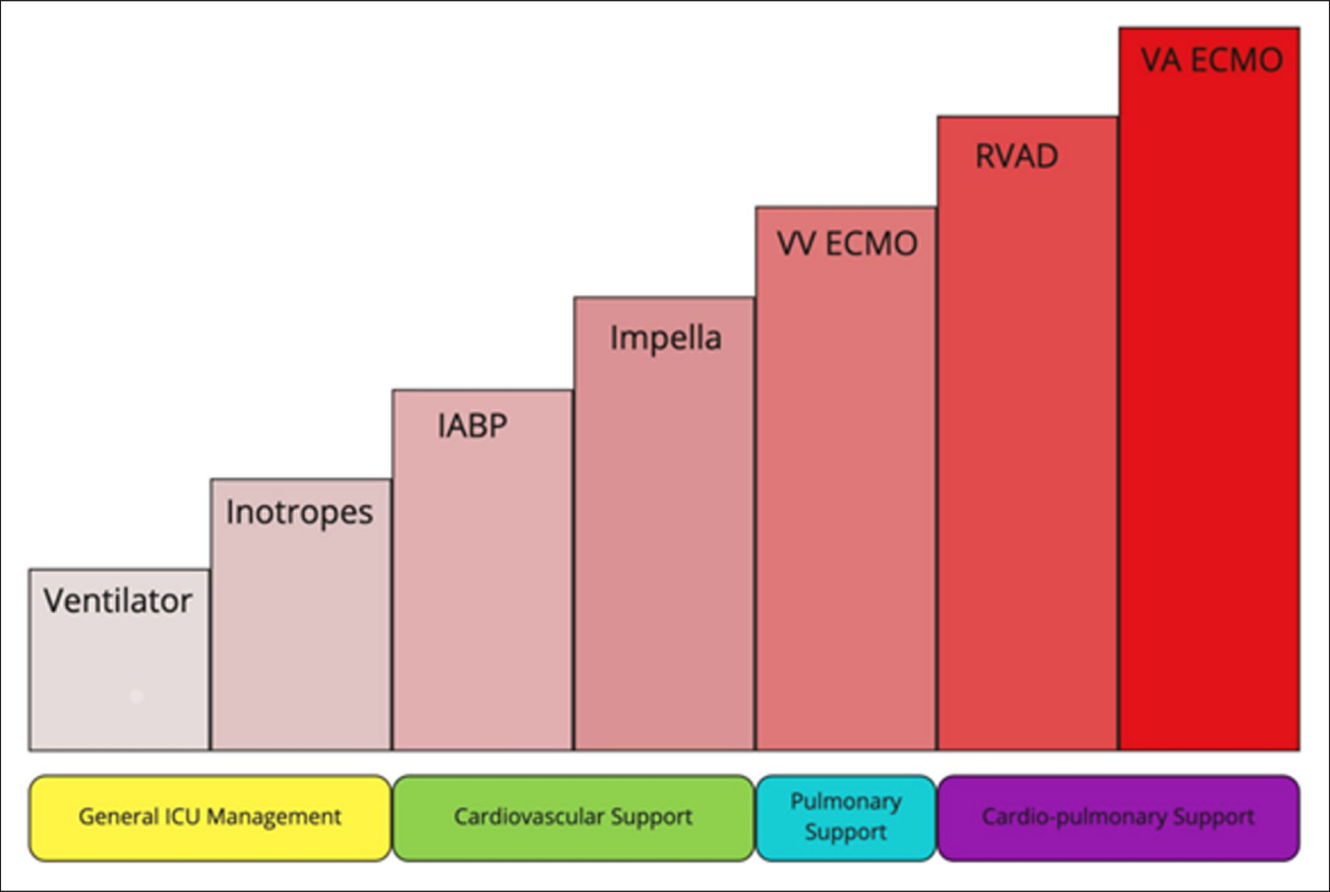
Spectrum of care in COVID-19 and Acute Circulatory Collapse. IABP: intra-aortic balloon pump; VV ECMO: veno-venous extracorporeal membrane oxygenation; RVAD: right ventricular assist device; VA ECMO: veno-arterial extracorporeal membrane oxygenation

Multiple temporary MCS options exist for cardiogenic shock, including IABP, Impella, TandemHeart (LivaNova), and extracorporeal membrane oxygenation (ECMO) (***Table 2***). Some of these modalities primarily support the LV and are better suited for isolated cardiac dysfunction, while other modalities provide combined cardiopulmonary support (***Figure 3***). Though data are limited in cases with COVID-19–related shock, the prevalence of classic cardiogenic shock with low EF and low cardiac index in the absence of respiratory involvement appears to be low, about 1 in 9 patients according to one study.^19^ Moreover, respiratory failure with ARDS is the predominant manifestation with severe COVID-19 infection. Therefore, MCS in cases of refractory cardiopulmonary failure in severe COVID-19 infection is mainly ECMO, with concurrent use of Impella and IABP for unloading the ventricle.

**Table 2:** Overview of mechanical circulatory support (MCS) strategies. VA ECMO: veno-venous extracorporeal membrane oxygenation; RVAD: right ventricular assist device; VV ECMO: veno-venous extracorporeal membrane oxygenation; IABP: intra-aortic balloon pump; LV: left ventricle; RV: right ventricle; BiV: biventricular


	MCS STRATEGY	CANNULATION SITE		CONSIDERATIONS

		INLET	OUTLET	

**Cardiopulmonary support**	VA ECMO	Femoral/jugular veins (peripheral) Right atrium (central)	Femoral artery (peripheral) Aorta (central)	LV or BiV failure May need LV venting strategy

	RVAD	Right atrium Internal jugular vein	Pulmonary artery	Predominant RV failure Oxygenator for pulmonary support

**Pulmonary support**	VV ECMO	Right atrium Internal jugular vein	Right atrium Internal jugular vein	Isolated respiratory failure

**Cardiac support**	Impella	LV	Proximal aorta	Direct LV support

	IABP	N/A	N/A	Indirect LV support via decreased afterload and improved coronary blood flow


### ECMO

The two common types of ECMO are veno-venous (VV) ECMO and veno-arterial (VA) ECMO. VV ECMO provides pulmonary support by oxygenating blood drained from the venous system and returning blood back to the venous system—the inferior vena cava, internal jugular vein, or femoral vein. VA ECMO also drains blood from the venous system but returns it into the aorta or common femoral artery, bypassing both cardiac and pulmonary function. VV ECMO is useful in patients with acute respiratory failure despite mechanical ventilation, whereas VA ECMO is better for patients with concomitant pulmonary and circulatory collapse.^20^

ECMO cannulation can be central or peripheral, single or double, and have unique triple cannula configurations (VVA, VAV, VAPa) for use in advanced ECMO cases requiring additional off-loading or drainage.^21,22^ The site of cannulation presents specific risks: Femoral artery cannulation may cause acute limb ischemia, and carotid artery cannulation has a higher risk of large watershed cerebral infarction.^20^ Femoral artery cannulation is preferred in cardiogenic shock cases because it is less invasive and allows faster cannulation.^20^ Patients should be anticoagulated to mitigate the risk of cannula-associated thrombosis, but this anticoagulation causes major or severe bleeds in up to one-third of patients.^23^ Most importantly, increased afterload in VA ECMO may require unloading with IABP or Impella.

Patient selection is essential because ECMO is a highly capital- and labor-intensive modality. Therefore, judicious use requires management by specialized staff along with multidisciplinary team discussions with surgeons, intensivists, cardiologists, and pulmonologists. At Houston Methodist, decisions regarding ECMO are made in conjunction with surgeons, heart-failure cardiologists, and intensivists. Patient selection is based on age, comorbidities, severity of multiorgan failure based on scoring systems such as the Sequential Organ Failure Assessment (SOFA) Score, and overall prognosis.^24^ At our institution, we use exclusion criteria outlined in ***Table 3***.

**Table 3:** Absolute and relative contraindications to extracorporeal membrane oxygenation. SOFA: Sequential Organ Failure Assessment; VAD: ventricular assist device; DVT: deep vein thrombosis


ABSOLUTE CONTRAINDICATIONS	RELATIVE CONTRAINDICATIONS

Age > 80 years Irreversible multisystem organ failure SOFA Score > 11 Contraindication to anticoagulation Unrecoverable cardiac condition, not a candidate for VAD/transplant Active life-limiting condition such as disseminated malignancy Cardiac arrest with asystole persisting for over 30 minutes	Skin infection at the site of cannulation Evidence of DVT in bilateral femoral veins Severe peripheral vascular disease (risk of limb ischemia) Intracerebral hemorrhage or severe brain damage Intubated > 7 days Obesity


Use of ECMO in the initial wave of COVID-19 produced mixed results. In an analysis of the Extracorporeal Life Support Organization (ELSO) registry between January and May 2020, the cumulative incidence of in-hospital mortality 90 days after initiation of ECMO support in COVID-19 patients was 37.4% (95% CI, 34.4-40.4). The presence of circulatory support with VA or VV ECMO was associated with significantly higher in-hospital mortality (HR 1.89; 95% CI, 1.2-2.97), likely because ECMO candidates were more critically ill.^25,26,27^

Despite these challenging odds for patients with combined heart and lung involvement, ECMO is often the only robust modality that can serve as a bridge to recovery or transplant, particularly in young patients with few other comorbidities who otherwise have a high chance of survival. Also, selecting patients in acute circulatory collapse who would benefit most from ECMO is likely to improve outcomes relative to liberal or futile use.^24^

There is little evidence to suggest the optimal process of weaning patients off ECMO and decannulating.^28^ Since the indications for having VV and VA ECMO are different, so are the weaning processes. Regardless, no standard approach has been established, and the weaning off ECMO is performed by an expert on a case-to-case basis depending on etiology. In general, cardiac and pulmonary function and recovery should be assessed before beginning weaning trials.

In VV ECMO, signs of pulmonary recovery such as lung compliance and ventilatory settings should first be assessed. If the fraction of inspired oxygen (FiO2) can be reduced to 40% on a ventilator and at least 90% on ECMO, then the ECMO sweep rate should be weaned and eventually ECMO flow rate should be reduced to the minimum tolerable setting. Flow rates should not be lower than 1.5 L/min due to the increased risk of clotting.^28^ Once VV ECMO sweep and flow rate settings have been minimized, it is appropriate to proceed with decannulation if the patient remains hemodynamically stable with good oxygen saturation; otherwise maintain full ECMO support.^24^

In VA ECMO, a similar approach should be used, but LVEF should be > 30%, mean arterial pressure > 60 mm Hg, cardiac index > 2.4 L/min, central venous pressure < 18 mm Hg, and arterial oxygenation > 90% before a team considers weaning.^24^

Patients who are re-cannulated or re-intubated usually have poor outcomes.^29^ Short-term support on ECMO is associated with better outcomes because long-term ECMO support carries high risks for hemolysis, thrombocytopenia, thrombosis, infection, and neurological damage.^30^ Thus, a patient who is on ECMO support and has persistent bleeding due to anticoagulation can be considered for decannulation because continuing the anticoagulation drastically increases the risk of thrombosis and embolic stroke.

### Intra-Aortic Balloon Pump

An IABP consists of a dual-lumen vascular catheter that is inserted percutaneously through the common femoral artery and placed at the descending aorta. The balloon tip is positioned at the distal end of the catheter, about 2 cm below the left subclavian artery. The central lumen allows for aortic pressure transduction, and the other lumen provides synchronized pumping of the balloon with the cardiac cycle. IABP reduces myocardial oxygen demand by decreasing afterload and improves diastolic coronary artery perfusion.^31^

Although the IABP-SHOCK II (Intraaortic Balloon Pump in Cardiogenic Shock II) trial showed no 30-day or long-term mortality benefit from using IABP in patients with cardiogenic shock due to acute myocardial infarction, its role in other causes of cardiogenic shock remains under-investigated.^32^ In the current COVID-19 era, IABP has been extremely useful for patients with isolated cardiac involvement because of simple monitoring, lower complication rates, and the ability to implant and explant at the bedside with minimal provider exposure.^31^ In certain cases of VA ECMO, IABP is essential for unloading the LV. Important considerations include anticoagulation and acute limb ischemia.

### Impella

An Impella (Abiomed) is a microaxial pump that uses the Archimedes screw principle to provide forward flow intraventricularly. It is positioned across the aortic valve, where the catheter inlet is positioned in the LV and catheter outlet is positioned in the proximal aorta. Impella improves forward flow (cardiac index) by unloading the LV, reducing LV end diastolic pressure, LV wall stress, and myocardial oxygen demand.^31^ For biventricular support, a Bi-pella (biventricular Impella) approach could be considered.^33^

Similar to IABP, placement of an Impella for cardiogenic shock due to acute myocardial infarction has not demonstrated better recovery or improved mortality compared to IABP.^34,35,36^ However, Impella is often used for LV unloading while on ECMO support.^37^ However, it does not provide feasibility of bedside implantation like IABP, and placing an Impella requires extensive staff support in a catheterization lab, which increases the risk of exposure for COVID-19. There have been cases of intracardiac echocardiography uses for bedside Impella placement, but it has its own complications such as limited spatial resolution that may lead to blind aortic instrumentation and other related complications.^38^

## Special Considerations

### Fulminant Myocarditis

Fulminant myocarditis is the sudden and severe inflammation of cardiac muscle that leads to either cardiogenic shock or multiorgan failure due to severe LV systolic function. Fulminant myocarditis is a rare presentation of COVID-19, with a mortality between 40% to 70%.^39^ Diagnosis is typically made via endomyocardial biopsy, but cardiac magnetic resonance imaging (MRI) is an equally useful modality.^40^ However, multiple case reports highlight the need for prompt identification, and management necessitates MCS with VA ECMO and biventricular Impella.^39,41,42,43^

### General Treatment Strategies

Although specific therapeutic management of severe COVID-19 has been comprehensively evaluated elsewhere, a discussion of the evidence is still warranted. Remdesivir inhibits all human and animal coronaviruses in vitro and is recommended for patients with severe COVID-19 infection. Remdesivir should be started within 72 hours of a positive polymerase chain reaction test in hypoxic patients but shows no benefit in critically ill patients who require MCS. Dexamethasone remains the mainstay of treatment in hypoxic patients, although evidence suggests potential harm for patients who do not require oxygen. Several Emergency Use Authorizations have been issued by the US Food and Drug Administration for monoclonal antibodies that may show a benefit in severe and critical illness, including the aforementioned IL-6 inhibitor tocilizumab. Notably, tocilizumab can reverse myocardial stunning in cytokine-induced cardiomyopathy seen in COVID-19.^44^ Other monoclonal antibody therapies have been authorized, but most are indicated for post-exposure prophylaxis rather than the treatment of severe or critical COVID-19 pneumonia.

### Anticoagulation

The National Institutes of Health guidelines on antithrombotic therapy in COVID patients are conservative, which is reflected by the literature.^45^ The REMAP-CAP (Randomised, Embedded, Multi-factorial, Adaptive Platform Trial for Community-Acquired Pneumonia), ACTIV-4a (Anti-thrombotics for Adults Hospitalized With COVID-19), and ATTACC (Antithrombotic Therapy to Ameliorate Complications of COVID-19) investigators illustrated that therapeutic anticoagulation did not confer an advantage of hospital survival or ventilator-free days.^46^ Patients requiring anticoagulation prior to hospitalization should remain anticoagulated. Patients requiring anticoagulation for a documented submassive or massive pulmonary embolism should receive it, and normal venous thromboembolism prophylaxis precautions should be followed.

Generally, ICU patients do not require anticoagulation due to COVID-19 without obvious indication. However, patients on MCS often require anticoagulation given the high risk of critical limb ischemia that accompanies cannulation.^23^ Excessive and persistent bleeding should prompt decannulation.

## Conclusion

Acute circulatory collapse in COVID-19 infection is a serious complication with poor outcomes. Early recognition of depressed LV function and cardiogenic shock by echocardiography, cardiac MRI, and Swan-Ganz catheter assessment is critical. ICU management of hemodynamic function, fluid status, and blood pressure management is the standard protocol, but prompt medical management with inotropes or tocilizumab and mechanical support of acute circulatory collapse maximizes patient outcomes. IABP, Impella, and VV and VA ECMO all play a role in managing acute circulatory collapse. Risks include acute limb ischemia and major bleeding from systemic anticoagulation, and appropriate patient selection is essential.

## KEY POINTS

- Acute circulatory collapse is a rare, life-threatening, and high-mortality complication of COVID-19.
- A Swan-Ganz catheter is necessary for early recognition of cardiogenic shock.
- Echocardiography can reveal depressed left ventricular ejection fraction, and cardiac magnetic resonance imaging can be useful in identifying fulminant myocarditis.
- Early escalation to mechanical circulatory support is necessary to reduce mortality if medical management fails.
- Anticoagulation, cannulation site, and appropriate patient selection all influence patient survivability.
- Tocilizumab may play a role in reducing cytokine-mediated acute circulatory collapse.
